# Metabolic reprogramming of CCR2^+^ MHC II^high^ macrophage contributes to myocarditis induced by Doxorubicin

**DOI:** 10.3389/fphys.2026.1883296

**Published:** 2026-10-27

**Authors:** Yingxin Li, Huiwen Jiang, Jiawei Shi, Jiani Qiu, Junmin Zhang, Jing Liu, Yilan Zhou, Ying Bai, Jingrong Jiang, Qiaofeng Jin, Li Zhang, Haotian Gu, Mingxing Xie, Tianshu Liu, Jing Wang

**Affiliations:** 1 Department of Ultrasound Medicine, Union Hospital, Tongji Medical College, Huazhong University of Science and Technology, Wuhan, China; 2 Hubei Province Clinical Research Center for Medical Imaging, Wuhan, China; 3 Hubei Province Key Laboratory of Molecular Imaging, Wuhan, China; 4 Institute of Hematology, Union Hospital, Tongji Medical College, Huazhong University of Science and Technology, Wuhan, Hubei, China; 5 School of Cardiovascular and Metabolic Medicine & Sciences, King’s College London BHF Centre of Research Excellence, London, United Kingdom

**Keywords:** cardiotoxicity, CCR2+ MHC IIhigh macrophage, Doxorubicin, metabolic reprogramming, Shikonin

## Abstract

**Background:**

Doxorubicin-induced cardiotoxicity remains a major clinical challenge. The intricate immunomodulatory pathways responsible for cardiac damage remain incompletely defined. Cardiac CCR2^+^ MHC II^high^ macrophages have emerged as a critical subset contributing to inflammatory cardiovascular injury. However, the precise exacerbating role and mechanisms by which these macrophages contribute to doxorubicin-induced myocardial injury remain elusive. Metabolic reprogramming significantly influences the transition of macrophages to an inflammatory phenotype, and pyruvate kinase M2 (PKM2), a key rate-limiting enzyme of glucose metabolism, may be a possible regulatory node and a promising therapeutic target for intervention.

**Methods:**

Doxorubicin-induced cardiotoxicity model was established in C57BL6/J mice. This model was utilized to assess the dynamic change relationship between cardiac function, cardiac CCR2^+^ MHC II^high^ macrophages, and myocardial injury remodeling. Also, the crosstalk between macrophage phenotypic switching and cardiomyocyte damage was explored *in vitro*. We further investigated the role of PKM2 in CCR2^+^ macrophage metabolic reprogramming and the therapeutic potential of using Shikonin to inhibit PKM2 to ameliorate cardiac injury. Moreover, peripheral blood cells from patients receiving Doxorubicin chemotherapy were used to verify the changes in PKM2 in macrophages, which revealed the role of metabolic reprogramming in pro-inflammatory macrophages.

**Results:**

CCR2^+^ MHC II^high^ macrophages may play a crucial role in exacerbating sustained cardiotoxicity caused by Doxorubicin. *In vitro* studies have shown that PKM2 facilitates the transition of macrophages into a pro-inflammatory CCR2^+^ phenotype. The phosphorylation of PKM2 mechanistically contributes to the metabolic reprogramming of these pro-inflammatory CCR2^+^ macrophages. *In vivo* and *in vitro* experiments have demonstrated that pharmacological inhibition of PKM2 with Shikonin mitigates doxorubicin-induced cardiotoxicity.

**Conclusion:**

Our results reveal a pivotal metabolic regulator of cardiac CCR2^+^ MHC II^high^ macrophages through metabolic reprogramming. Targeting PKM2 could offer a novel approach for mitigating Doxorubicin-induced myocardial inflammatory damage. Exploring the impact of CCR2^+^ macrophages on exacerbating myocardial injury sheds new light on the interplay between aberrant immune response of macrophage and Doxorubicin-induced myocarditis.

## 1 Introduction

Doxorubicin (DOX) is one of the most potent antitumor anthracycline antibiotics, with a broad spectrum and widely employed to treat various types of cancers. However, its cumulative dose-dependent tissue damage remains a major clinical challenge, particularly severe early or late cardiotoxicity effects (D’Angelo et al., 2022). The primary contributor of DOX on cardiomyocytes is oxidative stress (Shi et al., 2023). However, interventions inhibiting oxidative stress have failed to reduce the incidence of chronic dilated cardiomyopathy in human (Zhang et al., 2012). Accumulating clinical evidence suggests that inflammation plays a crucial role in the pathogenesis of persistent myocardial injury (Ruparelia et al., 2017). Interestingly, the knowledge to address the cardiac inflammatory injury mediated by aberrant immune response is still unknown in current study (Chen et al., 2022; Bayer et al., 2024).

Increasing attention has been directed toward non-myocyte cardiac cells, including T cells (Bayer et al., 2024) and macrophages (Zhang et al., 2020), which may contribute to cardiomyocyte toxicity (Tocchetti et al., 2020). Recent studies suggest that macrophages aggravate cardiac injury during chronic DOX exposure (Gambardella et al., 2023). However, the specific macrophage subsets and their roles remain unclear. Given their heterogeneity, macrophages exhibit distinct functions in cardiovascular inflammation. C-C motif chemokine receptor 2 (CCR2) distinguishes infiltrating monocyte-derived macrophages from resident macrophages: CCR2^+^ macrophages are recruited from the circulation in response to injury, whereas CCR2^−^ macrophages are primarily tissue-resident cells derived from embryonic precursors (Steffens et al., 2022). These populations have distinct effects on cardiac remodeling through crosstalk with cardiomyocytes, with CCR2^+^ macrophages generally promoting inflammation, remodeling, and fibrosis, while CCR2^-^macrophages support tissue homeostasis and repair (Yang et al., 2025). Targeting CCR2^+^ macrophages has shown anti-inflammatory effects in heart transplantation (Vasudevan et al., 2023), atherosclerosis (Georgakis et al., 2022), autoimmune myocarditis (Liu T. et al., 2023), and myocardial ischemia (Ding et al., 2025). Nevertheless, the role of CCR2^+^ macrophages in DOX-induced myocardial injury remains poorly defined. Metabolic reprogramming regulates macrophage phenotype and function and may influence cardiac injury (Liu et al., 2021). DOX can induce mitochondrial dysfunction in macrophages, increasing their reliance on glycolysis and promoting metabolic reprogramming (Gambardella et al., 2023). However, the effects of this process on specific macrophage subsets remain unclear (Kang et al., 2024). Enhanced glycolysis may further increase oxidative stress and sustain inflammation (Kang et al., 2024). Pharmacological or genetic inhibition of glycolysis can disrupt macrophage signaling and macrophage–cardiomyocyte crosstalk, thereby reducing persistent inflammatory injury (Liu et al., 2025). In addition, pharmacological inhibition of CCR2 signaling has been reported to affect glycolytic enzyme expression and activity in breast cancer cells (Acevedo et al., 2022). Thus, targeting glycolytic metabolism in CCR2^+^ macrophages may represent a potential therapeutic strategy for DOX-induced cardiotoxicity.Glucose metabolism is important for macrophage pro-inflammatory function (Han et al., 2025). Pyruvate kinase is one of the key enzymes in the glycolytic pathway. In sepsis, pyruvate kinase M2 (PKM2) interacts with hypoxia-inducible factor 1α (HIF1α) and activates the HIF-1α-dependent transcription of enzymes necessary for aerobic glycolysis in macrophages (Yang et al., 2014). Similarly, a potential PKM2 inhibitor, Shikonin, reduces serum lactate and HMGB1 levels, and protects mice from lethal endotoxemia and sepsis (O'Neill and Pearce, 2016). Emerging evidence demonstrates that PKM2 expression in macrophage from blood is increased in coronary heart disease patients, suggesting that PKM2 may be involved in crosstalk between macrophage and cardiovascular injury (Li et al., 2020). Shikonin (SHI), a naphthoquinone compound, is the main bioactive component from Zicao. Currently, there is an increasing interest in SHI due to its multiple pharmacological functions, such as anticancer, antiviral, antioxidant and anti-inflammatory properties (Liu F. Y. et al., 2023). Inhibition of PKM2 by SHI impedes TGF-β1 expression by repressing histone acetylation to alleviate renal fibrosis (Xiang et al., 2025). However, whether PKM2 regulates CCR2^+^ macrophage pro-inflammatory effect through glycolytic metabolism and thereby mediates the pathogenesis of cardiotoxicity is still unclear. The therapeutic potential of SHI-mediated inhibition of PKM2 expression in CCR2^+^ macrophages to disrupt their crosstalk with cardiomyocytes in DOX-induced cardiotoxicity remains to be investigated.

Here, we investigated the potential association between PKM2-related metabolic reprogramming and the inflammatory phenotype of CCR2^+^ macrophages in DOX-induced cardiotoxicity, and explored the effects of pharmacological PKM2 modulation with Shikonin. We first established the presence of crosstalk between CCR2^+^ macrophages and cardiomyocytes, which intensifies the inflammatory injury in DOX-induced cardiotoxicity. Subsequently, we examined the relationship between PKM2 expression or phosphorylation and DOX-associated glycolytic changes in CCR2^+^ macrophages. Finally, we evaluated whether Shikonin treatment was associated with attenuation of DOX-induced cardiotoxicity and macrophage metabolic changes.

## 2 Materials and methods

### 2.1 Model of Doxorubicin-induced cardiotoxicity and intervention protocol

Male C57BL6/J mice 8 weeks old were purchased from SPF Biotechnology Co., Ltd. (Beijing, China). The mice were housed in a pathogen-free room under a 12 h light/12 h dark environment at 23 °C at Tongji Medical College, Huazhong University of Science and Technology, China. The animal experiments were approved by the Institutional Animal Care and Use Committee at Tongji Medical College, Huazhong University of Science and Technology, China [(2021) IACUC 2438]. The animal experiments followed the guidelines from Directive 2010/63/EU of the European Parliament on the protection of animals used for scientific purposes. Following acclimatization, animals were stratified by body weight and divided into the vehicle control group (CON) and the Doxorubicin treatment group (DOX) via stratified randomization using a random number table. DOX (Med Chem Express. cat. nos. HY-15142) was dissolved in saline. Mice in the CON group received the following vehicles: equal volume intraperitoneal injection of normal saline (NS). Mice in the DOX group received DOX via an intraperitoneal injection (5 mg/kg body weight) once weekly for 3 weeks (cumulative dosage 15 mg/kg). To observe the change of infiltrating macrophages in myocardial tissue with the cumulative injury of DOX mimicking clinical situation, mice were executed and evaluated on day 7, 14 and 21. Finally, Shikonin (SHI; purity >98%) was administered by gavage at a dose of 2 or 5 mg/kg/day from day 0 to day 21. To assess the effects of SHI in the absence of DOX exposure, an additional SHI-only group received SHI at 5 mg/kg/day by gavage for 21 consecutive days, without DOX administration. The vehicle control group received the corresponding vehicle according to the same schedule. The SHI-only group was evaluated for the cardiac and systemic parameters included in the corresponding experiments.

In a subset of negative control experiments, DOX-treated mice were further randomly assigned to receive the selective small-molecule CCR2 antagonist INCB3344 (30 mg/kg per day. Med Chem Express. cat. nos. HY-50674) or vehicle (10% DMSO/0.9% carboxymethylcellulose) via daily intraperitoneal injections commencing 10 days after first injection of DOX and continuing until the end of the 21-day treatment period. To clarify the role of macrophages in accelerating DOX-induced cardiotoxicity, macrophage depletion was performed by Clodronate liposome (CL) treatment. Clodronate-loaded liposomes were purchased from LIPOSOMA (CP-005-005). For macrophage depletion, liposome (50 mg/kg) were injected intravenously into mice every 2 days lasting for 3 weeks after the first injection of DOX. DOX was administrated for 3 weeks together with the liposome treatment. Mice were examined on day 21.

### 2.2 Patient

Blood samples were obtained from patients receiving DOX chemotherapy and healthy donors. All investigations involving humans would have been performed in accordance with the revised Declaration of Helsinki (2008) (59th WMA General Assembly, Seoul, Republic of Korea, October 2008). Blood samples were drawn from all subjects prior to leukocytosis treatment. Information for all participants is provided in **Supplementary Table 1**. The flow cytometry gating strategy is shown in **Supplementary Figure 1**. Patients with a confirmed diagnosis of diffuse large B-cell lymphoma (DLBCL) who received doxorubicin-containing R-CHOP chemotherapy were eligible for the exploratory clinical analysis if a peripheral-blood sample was available for immunological assessment. Patients were excluded if they had a history of heart failure, active infection, autoimmune disease, clinically significant hepatic or renal dysfunction, or concomitant treatment with other immunomodulatory agents (**Supplementary Table 2**). These criteria were used to minimize the potential effects of pre-existing cardiac disease, systemic infection, immune dysregulation, organ dysfunction, and concomitant immunomodulatory therapy on immune-cell composition and cardiac-related measurements.

The human cohort was exploratory, and no formal *a priori* sample-size calculation was performed. The final sample size was determined by the number of eligible patients and available biospecimens during the study period. Demographic and clinical characteristics available for the cohort are summarized in **Supplementary Table 1**.

### 2.3 RNA sequencing dataset analysis

The dataset GSE226114 was re-analyzed using the method described in the original literature (Luo et al., 2023). Briefly, Gene Set Enrichment Analysis (GSEA) enrichment analysis was used to analyze the effect of synergistic gene changes on phenotypic changes in the WT and DOX groups in terms of chemokine receptors and ligands. The heat map shows the changes in chemokine ligand receptors and genes involved in glycolysis.

### 2.4 Echocardiography

Transthoracic echocardiography image acquisition and analysis were conducted under a full blinded protocol. Two independent investigators were tasked respectively with image acquisition and cardiac functional parameter quantification, and both were blinded to the group assignment of animals. All images were anonymized via random numerical coding after removal of all group identifiers. Key cardiac functional indices were measured in triplicate and averaged. Echocardiography was performed in mice using a Vevo 3100 instrument (Fujifilm Visual Sonics, Toronto, Canada) with a transducer MS400 (30 MHz). Mice were anaesthetized with 1.5% isoflurane, and two-dimensional echocardiographic views of the mid-ventricular short axis and parasternal long axis were obtained. Cardiac functions, such as left ventricular (LV) ejection fraction (EF) and fractional shorting (FS), were calculated using Vevo 3100 software.

### 2.5 Histological examination

To assess myocardial inflammation, hearts were sectioned transversely and stained with hematoxylin and eosin (H&E). All stained sections were assigned random codes and scored independently by two pathologists blinded to group allocation. Five high-power fields were randomly selected per section for evaluation, with the final inflammation score derived from the mean of the five fields. The inflammation score was as follows: grade 0, no inflammatory infiltrates; grade 1, < 25% of a cross section involved; grade 2, 25–50% of a cross section involved; grade 3, 50–75% of a cross section involved; > 75% of a cross section involved (Quah et al., 2007).

### 2.6 Cardiac infiltrating cell isolation and Flow cytometry analysis

Cardiac-infiltrating cells were isolated from mice hearts using minor modifications of the method described previously (Pinto et al., 2013). In brief, hearts were collected and perfused with HEPES buffer (20 mM HEPES, 130 mM NaCl, 3 mM KCl, 1 mM NaH2PO4, 4.5 mM glucose, pH=7.4) and then minced and digested in 0.1% collagenase B solution (C6885, Sigma, USA; 1 mg/ml in HEPES buffer) at 37 °C. The digested cell suspension was then filtered through 40 μm cell strainers to prepare a single-cell suspension. The cardiac lymphocytes isolated by density gradient centrifugation were cultured in a complete RPMI-1640 medium with 10% fetal bovine serum. The number of immune cells extracted is much less than that from the spleen. Therefore, according to the actual number of cells isolated, we will combine the hearts of two mice to ensure that there is enough number of similar suspended single cells for flow cytometry detection each time, which is conducive to subsequent statistical analysis and guarantee the quality of experimental results. The resulting single-cell suspension isolated from the cardiac and spleen was immune-stained with various combinations of fluorescing antibodies. After sample data collection was completed, gating and quantitative analysis were performed by a single independent investigator blinded to group allocation under a standardized gating strategy. Cardiac and splenic immune cell subsets gating strategy was shown in **Supplementary Figure 2** and **3**. Details are in **Supplementary Table 3**.

### 2.7 Quantitative real-time PCR analysis

Total messenger RNA was extracted using Trizol ^®^reagent (Invitrogen, USA) and converted into cDNA using PrimeScript RT Reagent Kit (Takara Biotechnology, Japan). Real-time PCR analyses were performed on a Bio-rad CXF CONNECT Detector system using SYBR green master mix (Takara Biotechnology, Japan). β-actin was used as the internal reference. Details are **Supplementary Table 4**.

### 2.8 TUNEL assay

RAW264.7 and H9C2 cell apoptosis was determined by terminal deoxynucleotidyl transferase dUTP nick-end labeling (TUNEL) staining by TUNEL Apoptosis Assay Kit with fluorescein (Beyotime, China) following the manufacturer’s instructions. Green fluorescein staining was used to identify apoptotic nuclei, while DAPI stained total nuclei. The apoptotic index was expressed as the percentage of apoptotic nuclei to total nuclei.

### 2.9 Cell culture and *in vitro* experimental protocols

RAW264.7 and H9C2 cells were provided by the cell bank of the Chinese Academy of Sciences (China). The cells were cultured in Dulbecco’s Modified Eagle Medium (DMEM) (high glucose) medium (Gibco, United States) supplemented with 10% fetal bovine serum (FBS) (Gibco, United States) and 1% of penicillin-streptomycin (Gibco, United States) in a humidified atmosphere of 5% CO2 at 37 °C. Cells were maintained in a proliferative state (i.e., undifferentiated state). To induce inflammatory injury, 0.5 µM of DOX was added to the cells culture 24 h. For PKM2 inhibitor experiments, cultures were co-incubated by 0.5 µM DOX for 24 h after the addition of SHI (5 µM, 2 h).

### 2.10 Glycolysis analysis using the Seahorse XF platform

Agilent XFe24 Extracellular Flux Analyzer was used to analyze extracellular acidification rates (ECAR) and oxygen consumption rates (OCR) of macrophages. A total of 50–000 macrophages were seeded in XFe 24-well cell culture microplates and allowed to recover for 24 h. Purified cell populations were washed in Seahorse XF DMEM supplemented with 1 mM sodium pyruvate, 2 mM L-glutamine, and 10 mM glucose. The medium was then replaced with Seahorse XF DMEM. Cells were allowed to adhere for 1 h at 37 °C in the absence of CO2. Finally, the cells were transferred to the XFe24 Flux Analyzer to measure cellular OCR and ECAR in accordance with the guidelines of the manufacturer. 1.5 μM oligomycin, 1μM FCCP, and 0.5 μM rotenone/antimycin A were injected sequentially into the microplates for the mitochondrial stress test. For the glycolysis rate assay, 0.5 μM rotenone/antimycin A and 50 mM 2-DG were injected sequentially into the microplates.

### 2.11 Western blotting analysis

Protein extracts of cells or tissue were prepared in lysis buffer containing a protease inhibitor and phosphatase inhibitor. Proteins were resolved on 12.5% sodium dodecyl sulfatepolyacrylamide gel electrophoresis (SDS-PAGE) gels and then immunoblotted to nitrocellulose membranes. After being blocked in Tris buffered saline with Tween 20 with 5% skim-milk for 1h at RT, the membranes were incubated with primary antibodies against P-STAT3 (Tyr705), STAT3, P-PKM2(Ser37), PKM2 or β-actin (**Supplementary Table 5**) at 4 °C overnight, and then washed and further incubated with secondary HRP-conjugated antibody for 2 h at room temperature. The membranes were finally washed.

### 2.12 Transmission electron microscopy

The RAW264.7 were fixed with 2.0% glutaraldehyde in 0.1 M sodium cacodylate buffer, pH 7.4, and then post-fixed in 1% osmium tetroxide, dehydrated in ethanol, and embedded in epon. Ultrathin sections of cells were collected on formvar-coated grids and were stained with uranil acetate and lead citrate. The samples were examined with a FEI Tecnai G2 20TWIN Transmission Electron Microscope operated at 200 KV.

### 2.13 CCK8

H9C2 and RAW264.7 cells were first counted and seeded in a 96-well cell culture plate (Corning Inc.). Then, after incubation at 37 °C in a humidified atmosphere with 5% CO2 for 24 h, the culture medium was replaced by a series of concentrations of SHI diluted with the corresponding culture fluid. Five replicates were made for each measurement, and the time of co-incubation was determined by the efficiency of each drug. Finally, 10 μL of the CCK-8 reagent (Med Chem Express Ltd.) was added into each well after 48h and OD at 450 nm was measured using a multifunction microplate reader (Infinite M200 Pro, Tecan) after incubation for 2 h at 37 °C. The percentage each concentration accounted for of the control was presented as cell viability.

### 2.14 Statistical analysis

Statistical analyses were performed using GraphPad Prism 8.0 (GraphPad Software, San Diego, CA, USA). This study was designed as an exploratory, mechanism-oriented investigation, and no formal *a priori* power calculation was performed. Sample sizes were determined based on previous studies using comparable experimental models, preliminary observations, experimental feasibility, the number of cells required for flow-cytometric analyses, and animal-welfare considerations. The sample size for each experiment is indicated in the corresponding figure legends.

An individual mouse or an independent cell-culture experiment was considered one biological replicate unless otherwise specified. For cardiac immune-cell analyses, hearts from two mice were pooled to obtain sufficient numbers of cells for flow-cytometric analysis. Therefore, each pooled sample was considered one biological replicate for statistical analysis. Technical replicate wells were averaged before statistical testing and were not treated as independent biological observations.

Formal normality testing was not systematically performed for every dataset because several experiments included relatively small sample sizes, which limits the reliability and interpretability of formal normality tests. For two-group comparisons, an unpaired two-tailed Student’s t-test was used where appropriate. Comparisons among more than two groups were performed using one-way analysis of variance (ANOVA), followed by Tukey’s multiple-comparisons test for *post hoc* pairwise comparisons. Tukey’s adjustment was applied within each ANOVA analysis. No global multiplicity correction was applied across all independent endpoints and figures because the analyses were exploratory and involved multiple mechanistic outcomes assessed in separate experimental settings. Data are presented as mean ± standard deviation (SD). All statistical tests were two-sided, and P < 0.05 was considered statistically significant.

## 3 Result

### 3.1 CCR2^+^ macrophage may have important synergistic effect to persistent cardiotoxicity during Doxorubicin chemotherapy in human

Cardiac macrophages represent a heterogeneous population of cells that can be divided into two major subsets with differing origins and functions: cardiac resident anti-inflammatory macrophages characterized by CCR2^-^/TIMD4^+^ and monocyte derived CCR2^+^ pro-inflammatory macrophages. CCR2^+^ macrophages play decisive roles in myocardial inflammatory injury (Zaman et al., 2021). In order to understand the changes of CCR2^+^ macrophage during myocarditis induced by DOX, we re-analyzed the bulk RNA-sequencing data of mice hearts from Ge et al (Luo et al., 2023), which consisted of all immune cells infiltrated into the heart. CCR2 had significant increased expression accompanied with decreased expression of TIMD4 (**Figure 1A**). GSEA enrichment analysis showed that the chemokine pathway was enriched in the DOX group (**Figure 1B**). Chemokines including CCL2, CCL7 and CXCL10, which induced CCR2^+^ macrophages to infiltrate into myocardial tissue, were increased (**Figure 1C**). In the presence of DOX, myocardial injury was evident.

**Figure 1:**
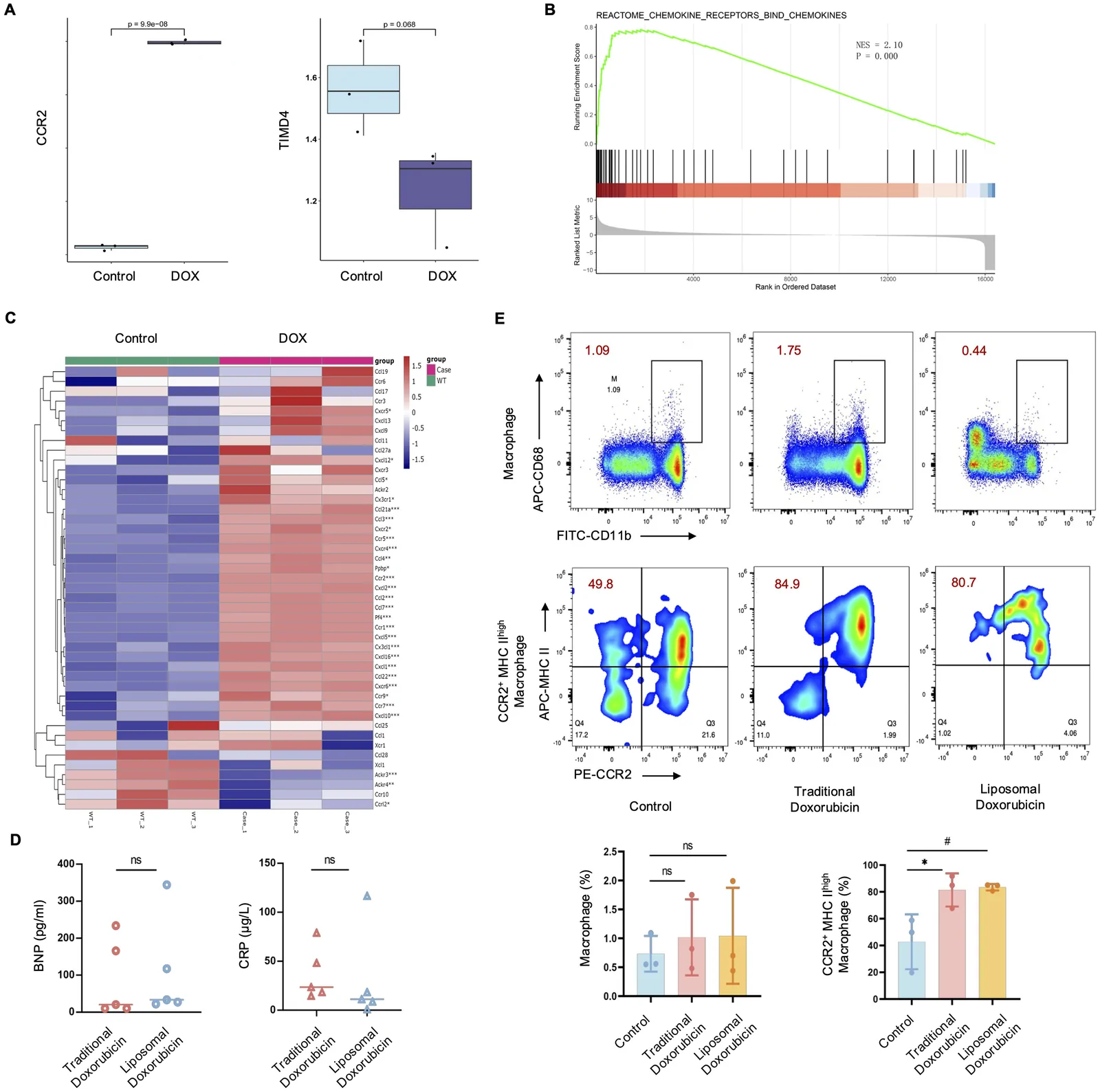
The characteristics of circulating CCR2^+^ macrophages in patients receiving Doxorubicin (DOX) chemotherapy combined with re-analysis of mouse RNA sequencing reveal the critical role of CCR2^+^ macrophages in myocardial inflammatory injury. **(A-C)** Re-analysis of mouse RNA sequencing of Doxorubicin myocardial injury including markers of resident macrophage (TIMD4) and pro-inflammatory macrophage (CCR2) **(A)**. GSEA enrichment analysis **(B)** and heat map **(C)** showed chemokines CCL2, CCL7 and CXCL10 were enriched in the DOX group. **(D)** Exploratory comparison of BNP and CRP levels between the traditional and liposomal doxorubicin groups (*n* = 5). No statistically significant differences were observed between the groups. These data were not compared with healthy-control values or clinical reference ranges. **(E)** Representative scatter plots of CCR2^+^ MHC II^high^ macrophage in the peripheral blood from healthy group and patients receiving Doxorubicin chemotherapy. (*n* = 3). Data were shown as mean ± SD, Student’s t-test for two-group comparison and one-way analysis of variance, followed by Tukey’s multiple comparison tests for comparison of multiple groups. *ns*, not statistically significant (*P > 0.05*). ^*^P < 0.05 represents control vs. traditional Doxorubicin chemotherapy. *^#^P < 0.05* represents control vs. liposomal Doxorubicin chemotherapy.

To alleviate the side effects of Doxorubicin chemotherapy, liposome-based dosage forms have been used clinically, but whether they could alleviate myocardial inflammatory damage is not clear. To explore the possibility of sustained myocardial damage by pro-inflammatory macrophages, we evaluated outcomes with a specific focus on changes in peripheral blood macrophages of 10 patients with diffuse large B-cell lymphoma (DLBCL). All enrolled patients received the standard R-CHOP regimen (rituximab, cyclophosphamide, doxorubicin/liposomal doxorubicin, vincristine, and prednisone) once every 3 weeks as a treatment cycle, with either conventional Doxorubicin (DOX) or liposomal Doxorubicin (Lip-DOX) as the anthracycline component. Concomitant administration of cyclophosphamide, vincristine, rituximab, and prednisone may exert independent modulatory effects on circulating monocyte and macrophage phenotypes, inflammatory biomarker levels, and cardiac function. Accordingly, clinical observations in human subjects represent correlative associations and are not exclusively attributable to doxorubicin alone. Echocardiography showed that left ventricular systolic function was preserved (EF > 50%), while transmitral flow was characterized by an E/A ratio < 1 in all enrolled patients, regardless of DOX chemotherapy regimen. Some patients had pericardial effusion and non-specific ECG abnormalities (**Supplementary Table 1**). BNP and CRP levels were measured in the traditional and liposomal doxorubicin groups, with no statistically significant differences observed between the groups (**Figure 1D**). The proportion and function of CCR2^+^ macrophages in peripheral blood were measured. Compared with healthy volunteers, the proportion of macrophages with antigen presenting function (CCR2^+^/MHC II^high^) in the peripheral blood of patients treated with Doxorubicin chemotherapy was significantly increased (**Figure 1E**). Therefore, regardless of the formulation, the cumulative use of Doxorubicin leading to abnormal immune responses in CCR2^+^ MHC II^high^ macrophages still has a potential risk of myocardial injury. Notably, these findings are based on a small-sample exploratory analysis and need to be verified in a larger cohort study. Given the limited sample size, the absence of age matching, and the small number of healthy controls, these human comparisons were considered exploratory. The data were used to assess consistency with the experimental findings and to generate hypotheses, rather than to establish definitive treatment effects or age-independent clinical associations.

### 3.2 The dynamic change characteristics of CCR2^+^ macrophage contributes to cumulative Doxorubicin-induced cardiotoxicity

To understand the characteristics between the infiltration of macrophages in myocardial tissue and the cumulative dose of Doxorubicin, the dynamic changes were analyzed. According to previous studies, we conducted the study at different time points of 7 days, 14 days and 21 days. We found that the body weight of the mice gradually decreased as the dose of the doxorubicin increased (**Figure 2A**). We then assessed the cardiac function of each group. Echocardiography showed that EF and FS were decreased gradually, accompanied by thinning of the ventricular wall in left ventricular anterior wall (LVAW) (**Figure 2B**; **Supplementary Table 6**). In addition, flow cytometry revealed that the proportion of macrophages (F4/80^+^/CD11b^+^) in the spleen and heart continued to increase on day 7 after Doxorubicin injection, but did not show change significantly in the subsequent time point. However, CCR2^+^ macrophages (F4/80^+^/CD11b^+^/CCR2^+^/MHC II^high^) in the heart, which represents the function of antigen presentation, gradually increased with the exposure time in a dose-dpendent manner. Among them, the mean fluorescence intensity of MerKT, which represents anti-inflammatory repair function of resident macrophages, was reduced with dose accumulation (**Figure 2C**). Consistent with these results, H&E and TUNEL staining also showed persistent myocardial inflammation and apoptosis (**Figures 2D, E**). The above suggested that cardiac CCR2^+^ macrophages may be a key regulator involved in the coordination between myocardial inflammatory necrosis and left ventricular systolic dysfunction caused by dose accumulation (**Figure 2F**).

**Figure 2:**
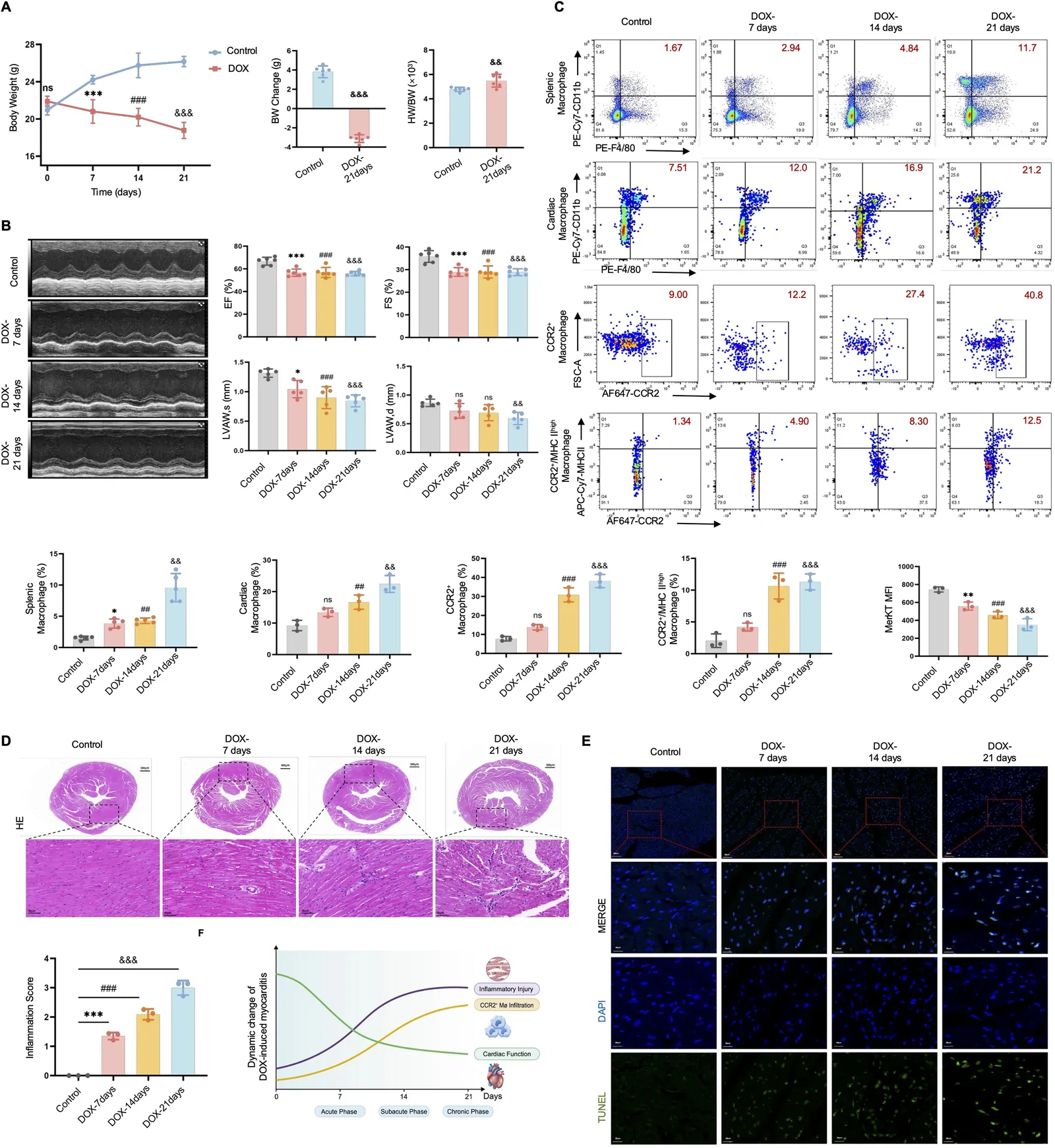
The dynamic changes of CCR2^+^ macrophage infiltrated in myocardium contributes to cumulative Doxorubicin-induced cardiotoxicity. **(A)** Dynamic body weight of DOX treated mice in different timepoints. **(B)** M-mode echocardiography images, quantification of EF and FS were shown to evaluate the cardiac function of DOX mice. **(C)** Representative FACS quantification of splenic and cardiac macrophage from DOX treated mice at day 0, 7, 14, 21. *n* = 5 mice per group for splenic macrophages; *n* = 3 pooled samples (2 hearts per sample) per group for cardiac macrophage subsets (F4/80^+^CD11b^+^, CCR2^+^, CCR2^+^ MHCII^high^, MerTK). **(D)** H&E staining for the degree of inflammatory cell infiltration of DOX hearts. Scale: 500 μm (top) and 50 μm (bottom). **(E)** TUNEL staining images of cardiac tissue stained with TUNEL-positive nuclei in green and DAPI in blue. Scale: 50 μm (top) and 20 μm (bottom) **(F)** Cardiac functional, pathological, and immunological correlations after Doxorubicin treatment were comprehensively analyzed to demonstrate the synergistic relationship. n = 3–5 per group. Data were shown as mean ± SD. Student’s t-test for two-group comparison and one-way analysis of variance, followed by Tukey’s multiple comparison tests for comparison of multiple groups. *ns*, not statistically significant (*P > 0.05*). ^*^P < 0.05,^**^P < 0.01 and ^***^P < 0.001 represent control vs. DOX-7 days. *^#^P < 0.05*, *^##^P < 0.01* and *^###^P < 0.001* represent control vs. DOX-14 days. *^&^P < 0.05*, *^&&^P < 0.01* and *^&&&^P < 0.001* represent control vs. DOX-21 days.

To explore the effect of CCR2^+^ macrophage on myocardial injury induced by Doxorubicin, macrophage depletion and CCR2 antibody neutralization were performed. Flow cytometry showed that Clodronate-loaded liposome treatment reduced the expression of CCR2^+^ (**Figure 3A**). Likewise, the percentage of CCR2^+^ macrophages in Doxorubicin-treated mice with receiving INCB3344 was significantly lower than that observed in the vehicle-treated group (**Figure 3B**). H&E staining in above experiments showed that the degree of myocardial inflammation was reduced than that in Doxorubicin group alone (**Figure 3C**). Based on the results of body weight and heart weight/body weight, treatment of macrophage depletion and CCR2 antibody neutralization had effect of remission (**Figure 3D**). *In vitro*, we found that RAW264.7 was able to be partially induced by DOX into pro-inflammatory macrophages expressing CCR2 (**Figure 3E**). To evaluate macrophage-dependent inflammatory injury of myocardial cell, we treated H9C2 cardiomyocyte directly with DOX (referred to as DOX) or with a conditioned supernatant of unstimulated macrophages (referred to as M-sup) and DOX-activated macrophages (referred to as DOX-M-sup). Both DOX and DOX-M-sup treatments reduced cell viability and increased apoptosis, but these effects were more obvious in Dox-M-sup treated cells (**Figure 3F**). It was manifested by lower expression of P-STAT3 (**Figure 3G**). This finding strongly suggested the existence of a synergy effect between cardiomyocyte apoptosis and CCR2^+^ macrophages switching induced by DOX.

**Figure 3:**
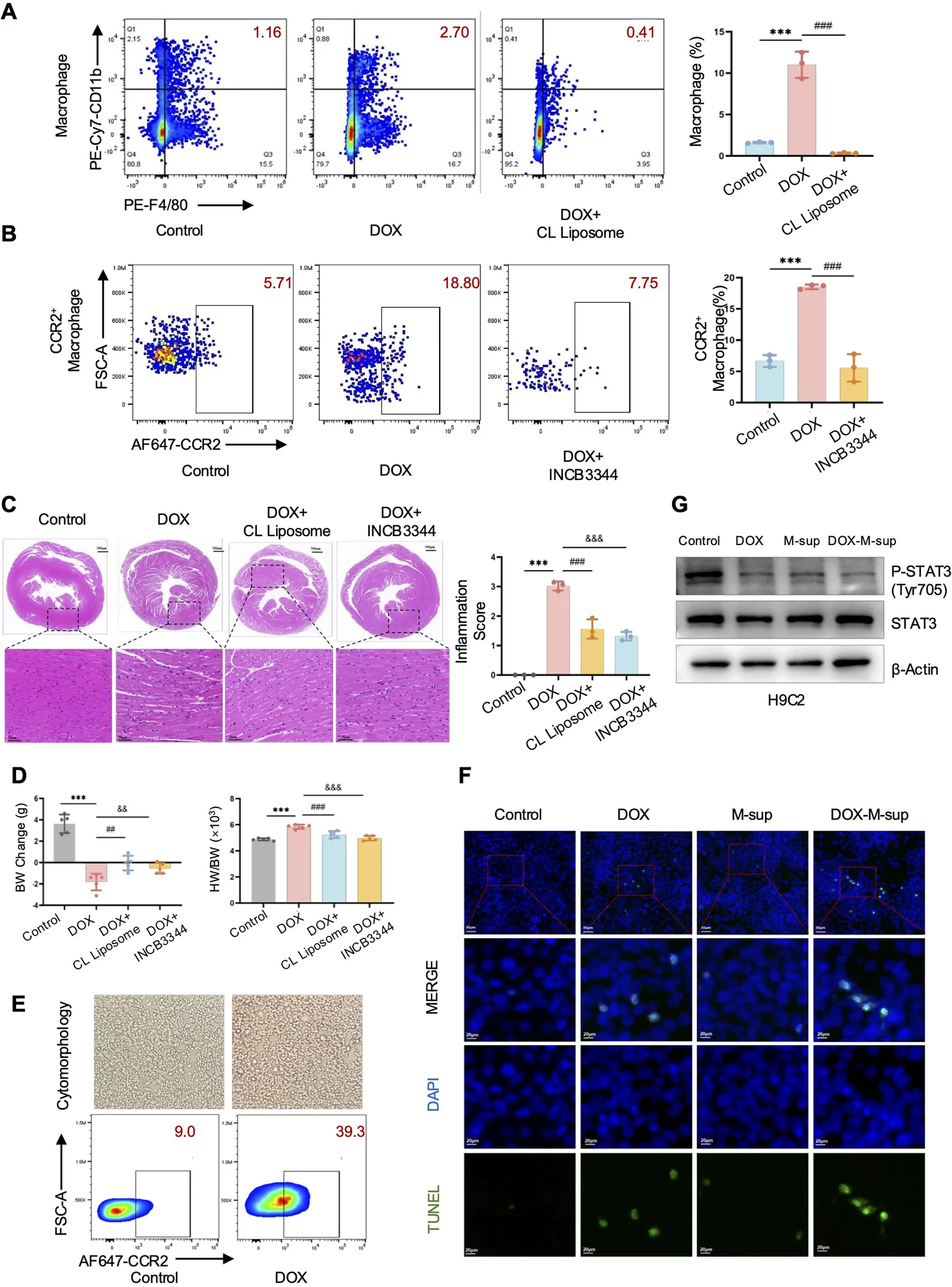
The crosstalk between CCR2^+^ macrophages and cardiomyocytes is critical for cardiotoxicity induced by Doxorubicin. **(A-C)** Representative FACS quantification of cardiac CCR2^+^ macrophage and H&E staining in macrophage depletion by Clodronate-loaded liposome (CL- Liposome) and selective small-molecule CCR2 antagonist INCB3344 in mice. *n* = 3 pooled samples (2 hearts per sample) per group for cardiac macrophage subsets. **(D)** Body weight and ratio of heart weight to body weight of each group (*n* = 5). **(E)** The proportion of RAW264.7 transformed to the CCR2^+^ phenotype after DOX treatment. **(F)** TUNEL staining images of cardiac tissue stained with TUNEL-positive nuclei in green and DAPI in blue. Scale: 50 μm (top) and 20 μm (bottom). **(G)** The supernatant of RAW264.7/DOX-treated RAW264.7 were reciprocally exchanged with H9C2, and the cells were harvested for Western blots. Data were shown as mean ± SD. One-way analysis of variance for comparison of multiple groups, following by Tukey’s multiple comparison tests. ^***^P < 0.001 represents control vs. DOX. *^##^P < 0.01* and *^###^P < 0.001* represent DOX vs. CL- Liposome. *^&&^P < 0.01* and *^&&&^P < 0.001* represent DOX vs. INCB3344.

### 3.3 Shikonin attenuates Doxorubicin-induced cardiotoxicity in association with modulation of PKM2-related pathways

Given the important role of metabolic reprogramming in pro-inflammatory macrophage, we reanalyzed changes in the key enzymes of glucose metabolism, and the heat map showed that many of them were significantly different, including GAPDH, HK (1/2/3), LDH (b/c), PFK (l/m/p), PKM (Nahrendorf and Swirski, 2016; Ryan et al., 2019). Since the above data are RNA sequencing of myocardial tissue, changes in single immune cells cannot be clarified. Further examination of DOX-induced RAW264.7 revealed the changes in different key enzymes of glucose metabolism and chemokines related to inflammatory responses. Among them, the transcription level of PKM2 was significantly increased under DOX conditions (**Figure 4A**). Further examination of DOX-induced RAW264.7 revealed changes in different key enzymes of glucose metabolism, and transcript level of PKM2 was significantly increased under DOX condition (**Figure 4B**). Meanwhile, DOX-treatment promoted the release of inflammatory factors IL-6 and IL-1β (**Figure 4C**). To further examine whether pharmacological modulation of PKM2-related pathways was associated with the pro-inflammatory transformation of CCR2^+^ macrophages, we treated the cells with Shikonin. Shikonin is a naturally occurring chemical compound derived from Lithospermum erythrorhizon. It is a naphthoquinone derivative, with diverse pharmacological properties, including anti-inflammatory, anti-tumor, antioxidant, and antimicrobial activities (**Figure 4D**) (Guo et al., 2023). First, we extracted peripheral blood cells from patients receiving doxorubicin-containing chemotherapy and observed that Shikonin treatment was associated with reduced PKM2 immunoreactivity in CCR2-positive cells (**Figure 4E**).

**Figure 4:**
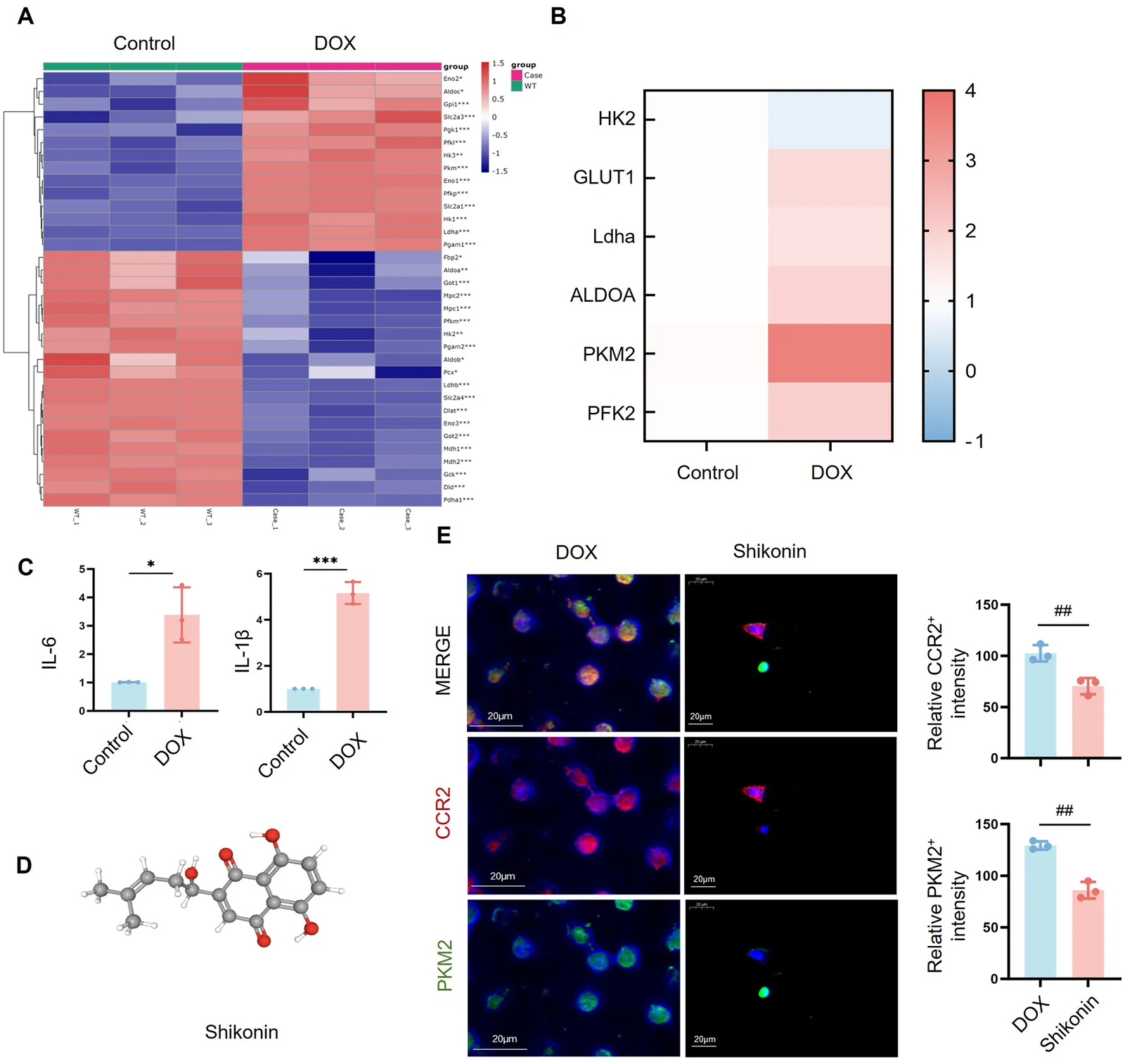
The expression of PKM2, a key enzyme in glucose metabolism, in macrophage under Doxorubicin condition. **(A)** Heat map of glycolytic genes in DOX-treated heart. **(B)** The gene expression level of different glycolysis-related enzymes in DOX-treated RAW264.7. **(C)** Level of IL-6 and IL-1β in control and DOX-treated RAW264.7. **(D)** Overall structure of Shikonin. **(E)** Immunofluorescence staining of PKM2 was performed in peripheral blood cells from patients receiving Doxorubicin and Shikonin treatment *in vitro*; nuclei were labelled with DAPI. The scale bar represents 20 µm. *n* = 3 per group. Data were shown as mean ± SD. Student’s t-test for two-group comparison. ^*^P < 0.05 and ^***^P < 0.001 represent control vs. DOX. *^#^P < 0.05* and *^##^P < 0.01* represent DOX vs. Shikonin.

Further studies are needed to clarify the impact of metabolic changes in CCR2^+^ macrophages on cardiotoxicity by Doxorubicin. iven the well-documented hepatotoxicity and narrow therapeutic window of shikonin in preclinical literature, we first evaluated the hepatic safety of shikonin monotherapy in healthy mice to validate the dosing regimen for subsequent *in vivo* experiments. Serum levels of aspartate aminotransferase (AST) and alanine aminotransferase (ALT), two classic biomarkers of hepatic injury, showed no statistically significant differences between the shikonin-treated group and the vehicle control group (**Supplementary Figure 4**), indicating that shikonin at the tested dose did not induce overt hepatic injury in mice. Following validation of the dosing regimen, We treated mice with Shikonin (2 or 5 mg/kg/day) after doxorubicin administration and evaluated the animals on day 21. To assess whether Shikonin itself affected cardiac function, body weight, cardiac morphology, or macrophage populations in the absence of doxorubicin, an additional SHI-only group received Shikonin at 5 mg/kg/day by gavage for 21 consecutive days. Compared with the DOX mice, Shikonin-treated mice exhibited a lower ratio of heart weight/body weight and body weight (**Figure 5A**). We then assessed the cardiac function of each group. Echocardiography showed that the EF and FS were decreased in the DOX group on Day 21, whereas the Shikonin-treated mice recovered these indices (**Figure 5B**; **Supplementary Table 4**). Consistent with these results, H&E and TUNEL staining also showed that both the cardiac inflammation area and apoptosis degree were reduced in the Shikonin group (**Figures 5C, D**). The increased plasma level of the cardiac damage marker CK-MB (**Figure 5E**) was found in DOX mice. However, it was significantly lower in Shikonin-treated DOX mice. In addition, we next explored the effect of Shikonin on CCR2^+^ macrophage. First, we found that Shikonin significantly reduced the ratio of splenic and cardiac macrophage (F4/80^+^/CD11b^+^). Furthermore, the infiltration of pro-inflammatory CCR2^+^ macrophage in heart were also reduced in Shikonin group (**Figure 5F**). Correspondingly, the expression of MHC II was substantially lower in the Shikonin group with higher MerKT expression (**Figure 5F**). These data further support the potential involvement of CCR2^+^ macrophages in cardiac dysfunction in response to DOX.

**Figure 5:**
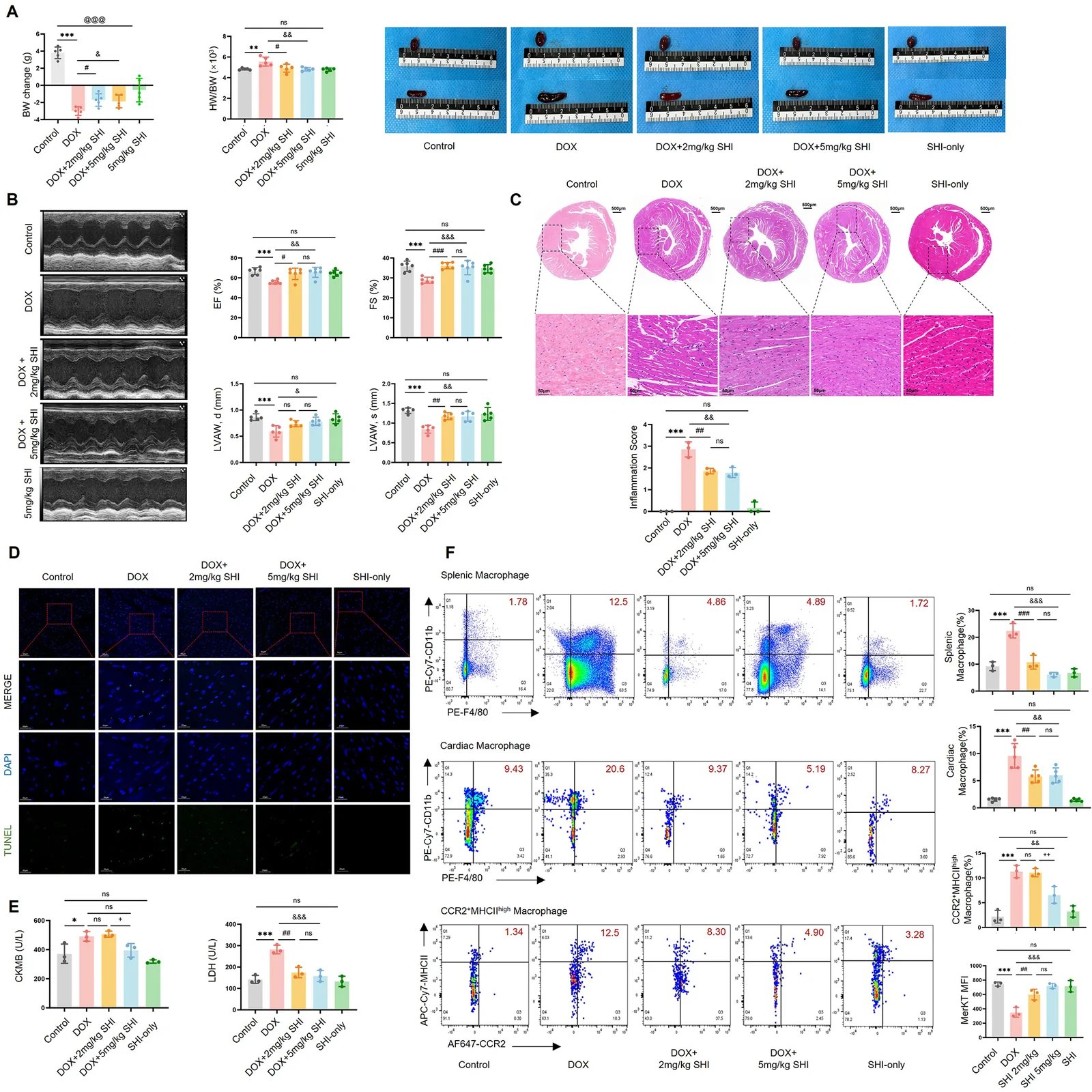
PKM2 inhibition reduces the severity of Doxorubicin-induced cardiotoxicty. **(A)** Body weight and HW/BW of each group. **(B)** M-mode echocardiography images, quantification of EF and FS were shown to evaluate the cardiac function of DOX or SHI treated mice. **(C)** H&E staining for the degree of inflammatory cell infiltration of hearts in different groups. Scale: 50 μm (top) and 20 μm (bottom). **(D)** TUNEL staining images of cardiac tissue stained with TUNEL-positive nuclei in green and DAPI in blue. Scale bars: 20 μm. **(E)** ELISA detected CK-MB and LDH levels in cardiac tissue from all experimental groups at day 21. **(F)** Representative FACS quantification of splenic macrophage and cardiac CCR2^+^ macrophages from DOX mice with or without SHI treatment at day 21. *n* = 5 mice per group for splenic macrophages; *n* = 3 pooled samples (2 hearts per sample) per group for cardiac macrophage subsets (F4/80^+^CD11b^+^, CCR2^+^ MHCII^high^, MerTK) Data were shown as mean ± SD. One-way analysis of variance for comparison of multiple groups, following by Tukey’s multiple comparison tests. *ns*, not statistically significant (*P > 0.05*). ^*^P < 0.05 represents control vs. DOX. ^**^P < 0.01 represents control vs. DOX. ^***^P < 0.001 represents control vs. DOX. *^#^P < 0.05* represents DOX vs. SHI (2mg/Kg). *^##^P < 0.01* represents DOX vs. SHI (2mg/Kg). *^###^P < 0.001* represents DOX vs. SHI (2mg/Kg). *^&^P < 0.05* represents DOX vs. SHI (5mg/Kg). *^&&^P < 0.01* represents DOX vs. SHI (5mg/Kg). *^&&&^P < 0.001* represents DOX vs. SHI (5mg/Kg). ^*^P < 0.05, ^**^P < 0.01 and ^***^P < 0.001 represent control vs. DOX.

### 3.4 Shikonin treatment is associated with reduced conversion of macrophages to a CCR2^+^ inflammatory phenotype *in vitro*

To further investigate the effects of macrophage PKM2 inhibition on DOX-conditioned cardiomyocytes *in vitro*, we performed the following compared or co-cultured experiments. First, the CCK8 assay showed macrophage and cardiomyocyte activity at different SHI concentrations. We selected a drug concentration of 2.5μm that is safe for both (**Figure 6A**). Flow cytometry revealed that the SHI group had lower expression of CCR2^+^ in DOX-induced RAW264.7 (**Figure 6B**). WB showed that SHI reduced the phosphorylation of PKM2 (**Figure 6C**). Notably, CCR2 and PKM2 co-localization showed that p-PKM2 fluorescence intensity was decreased more obviously than PKM2 in CCR2-labeled RAW264.7 of SHI treatment (**Figure 6D**). To investigate the effect of SHI-treated macrophages on cardiomyocyte inflammation, we treated H9C2 cardiomyocyte and Primary neonatal mouse cardiomyocytes (PNMCs) with a conditioned supernatant of SHI-cultured macrophages (referred to as M-SHI-sup) or SHI-cultured macrophages induced by DOX previously (referred to as DOX-M-SHI-sup). Compared with Dox-M-sup group, Dox-M-SHI-sup treatment increased cell viability and reduced apoptosis, and this is more obvious than the cells treated with SHI alone (**Figures 6E, F**; **Supplementary Figure 5**). These findings suggest that the cardioprotective effects of SHI may be partly mediated through modulation of activated macrophage-derived inflammatory signals, although direct effects on cardiomyocytes cannot be excluded.

**Figure 6:**
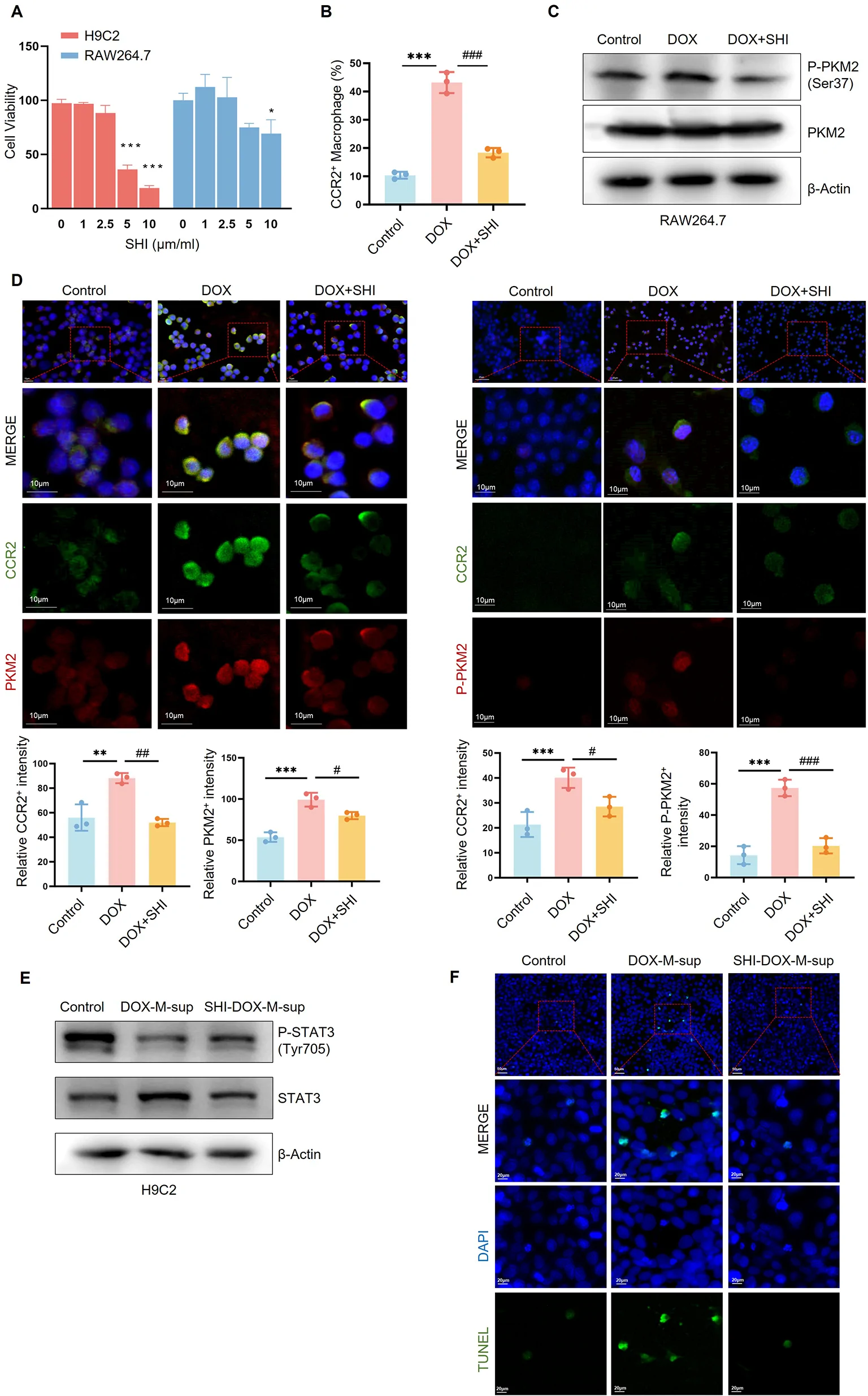
PKM2 promotes the phenotype transformation and pro-inflammatory function of CCR2^+^ macrophage *in vitro*. **(A)** CCK8 assay shows the activity of H9C2 and RAW264.7 under different Shikonin concentrations. **(B)** Representative FACS quantification of RAW264.7 transformed to the CCR2^+^ phenotype after DOX treatment with or without Shikonin. **(C)** Western blotting was performed to identify total and phosphorylated (Ser 37) levels of PKM2 in different group; β-actin was used as the loading control. **(D)** Immunofluorescence staining of intracellular PKM2 and p-PKM2 was performed in DOX treated RAW264.7 with or without Shikonin; nuclei were labelled with DAPI. The scale bar represents 10 µm. **(E)** The supernatant of RAW264.7, DOX-treated RAW264.7 and SHI-treated RAW264.7 were reciprocally exchanged with H9C2. Western blotting of H9C2 was performed to identify total phosphorylated (Tyr 705) levels of STAT3 in different group; β-actin was used as the loading control. **(F)** TUNEL staining images of cardiac tissue stained with TUNEL-positive nuclei in green and DAPI in blue. Scale bars: 20 or 50 μm. *n* = 3 per group. Data were shown as mean ± SD. One-way analysis of variance for comparison of multiple groups, following by Tukey’s multiple comparison tests. ^**^P < 0.01 and ^***^P < 0.001 represent control vs. DOX. *^#^P < 0.05*, *^##^P < 0.01* and *^###^P < 0.001* represent DOX vs. DOX+SHI.

### 3.5 Shikonin treatment is associated with metabolic reprogramming of pro-inflammatory CCR2^+^ macrophages

Macrophage metabolic reprogramming is essential to meet energy, and metabolite demands to deal with various critical environmental changes (Zhang et al., 2020). Under naive conditions, macrophages mainly rely on oxidative phosphorylation (OXPHOS) to generate ATP. When confronted with an inflammatory environment, macrophages tend to reprogram their energy production by relying on glycolysis. The switch to glycolytic metabolism in proinflammatory macrophages allows them to respond to the rapid, short-term bursts of activation required during the inflammatory phase. As metabolic reprogramming is critical to modulate macrophage polarization, we then examined how PKM2 inhibition affected metabolic reprogramming of CCR2^+^ macrophages. First, we hypothesize that this may be related to the inhibition of inflammatory signaling pathways in macrophages. SHI treatment could reverse the pro-inflammatory condition. We found that the production of mitochondrial reactive oxygen species was significantly decreased in SHI-treated proinflammatory macrophages but not in DOX stimulated-macrophages (**Figure 7A**). To explore the role of PKM2 in macrophage metabolic reprogramming, we measured the extracellular acidification rate (ECAR; **Figure 7B**) and oxygen consumption rate (OCR; **Figure 7C**), which are indicators of glycolytic capacity and OXPHOS, respectively. Inhibition of PKM2 significantly reduced glycolytic capacity and increased the mitochondrial respiration rate in proinflammatory macrophage. SHI altered ECAR in basal and maximum glycolysis, indicating the regulation of metabolic reprogramming in PKM2 inhibitory proinflammatory macrophages. Glycolytic metabolism uses glucose as fuel, which requires efficient glucose uptake. Therefore, we assessed glucose uptake by macrophages using the fluorescence-labeled glucose analog 2-NBDG. The glucose uptake capacity of the SHI-treated proinflammatory macrophages was remarkably decreased compared to DOX group (**Figure 7D**). In addition, mitochondrial damage was attenuated in each SHI-treated cell as observed by electron microscopy. The extent of mitochondrial swelling was reduced, and mitochondrial cristae was reduced but morphologically normal. These results suggest that SHI-treated macrophages have stable mitochondrial function (**Figure 7E**). These findings are consistent with an association between PKM2 phosphorylation and metabolic reprogramming in pro-inflammatory macrophages. However, whether PKM2 phosphorylation is causally required for this process will require validation using genetic loss-of-function, gain-of-function, or rescue approaches.

**Figure 7:**
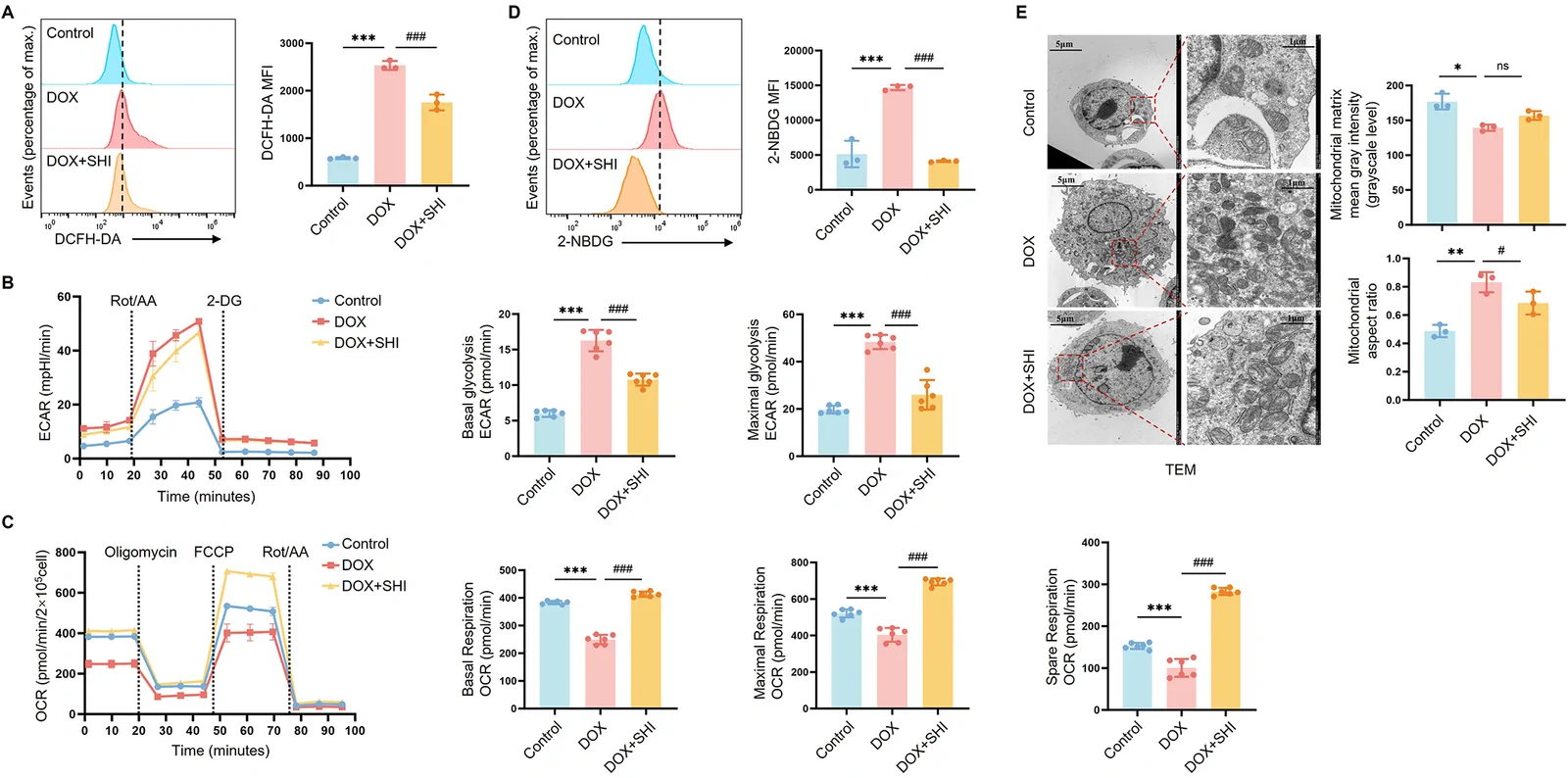
PKM2 contributes metabolic reprogramming of proinflammatory CCR2^+^ macrophages. **(A)** Representative reactive oxygen species flow cytometry histogram (left panel) and quantity chart (right panel) in control, proinflammatory and PKM2 inhibitory macrophages. *n* = 3 per group. **(B, C)** Seahorse analysis of ECAR and OCR in control, proinflammatory and PKM2 inhibitory macrophages. *n* = 6 per group. **(D)** Representative flow cytometry histogram (left panel) and quantity chart (right panel) of glucose uptake in control, proinflammatory and PKM2 inhibitory macrophages. *n* = 3 per group. **(E)** Representative mitochondria images in different groups. n = 3–5 per group. Data were shown as mean ± SD. One-way analysis of variance for comparison of multiple groups, following by Tukey’s multiple comparison tests. *ns*, not statistically significant (*P > 0.05*). ^*^P < 0.05, ^***^P < 0.01 and ^***^P < 0.001 represent control vs. DOX. *^#^P < 0.05* and *^###^P < 0.001* represents DOX vs. DOX+SHI.

## 4 Discussion

This study provides evidence that infiltrating CCR2^+^ macrophages are associated with the progression of doxorubicin (DOX)-induced cardiotoxicity. In DOX-exposed pro-inflammatory macrophages, PKM2 expression and phosphorylation were increased and accompanied by enhanced glycolytic activity. In our experimental models, Shikonin treatment reduced CCR2^+^ macrophage accumulation, glycolytic activity, inflammatory features, and cardiac injury parameters. However, because macrophage-specific PKM2 genetic manipulation and rescue experiments were not performed, these findings support an association between PKM2-related metabolic changes and the inflammatory phenotype of CCR2^+^ macrophages but do not establish PKM2-specific causality.

The human findings should be interpreted cautiously. All patients with diffuse large B-cell lymphoma (DLBCL) received doxorubicin-containing R-CHOP chemotherapy rather than DOX monotherapy. Therefore, the observed changes in CCR2^+^MHC II^high^ macrophages, B-type natriuretic peptide (BNP), C-reactive protein (CRP), and diastolic parameters may reflect the combined effects of doxorubicin, cyclophosphamide, vincristine, rituximab, prednisone, disease-related inflammation, and other clinical factors. Moreover, the healthy controls were younger than the patient cohort, which may have contributed to differences in immune-cell composition and diastolic function. Accordingly, these observations are associative and hypothesis-generating. Although consistent with the experimental findings, they cannot establish a DOX-specific or age-independent association or demonstrate that DOX alone caused the observed immune or cardiac changes.

The myocardium is particularly susceptible to DOX-induced injury, but the contribution of distinct macrophage subsets remains incompletely understood. In mice, myocardial infiltration by CCR2^+^ macrophages increased with cumulative DOX exposure and was accompanied by cardiomyocyte apoptosis, whereas impairment of left ventricular systolic function was detectable at an earlier stage. During sustained inflammation, circulating CCR2^+^ macrophages appeared to represent a predominant inflammatory macrophage population. Macrophage depletion and CCR2 blockade reduced myocardial inflammatory injury, while supernatant-transfer experiments showed that inflammation-activated macrophages enhanced cardiomyocyte apoptosis *in vitro*. These findings suggest that CCR2^+^ macrophages may aggravate cardiac injury through macrophage–cardiomyocyte crosstalk and that modulation of this macrophage phenotype may represent a potential therapeutic strategy.

Our results are broadly consistent with previous studies showing that macrophages contribute to DOX-induced cardiac injury, although the macrophage populations and mechanisms identified across studies are not identical. Cardiac resident macrophages have been reported to exert protective or reparative effects, whereas infiltrating inflammatory macrophages may amplify myocardial damage. These differences may reflect variations in macrophage origin, disease stage, DOX dosing regimen, tissue-sampling time, and phenotypic definitions. Our study focused on circulating monocyte-derived CCR2^+^ MHC II^high^ macrophages, which increased during cumulative DOX exposure and were associated with inflammatory myocardial injury. Thus, our findings complement rather than exclude a protective or reparative role for resident macrophages during other stages of DOX-induced cardiomyopathy. Their potential contribution, particularly during the chronic phase, requires further investigation.

Metabolic reprogramming in immune cells is influenced by oxygen availability, nutrient status, and immune signals (Sun et al., 2020). Pro-inflammatory macrophages commonly undergo a shift toward increased glucose uptake and aerobic glycolysis, often referred to as the Warburg effect, with reduced reliance on oxidative phosphorylation. DOX-induced mitochondrial injury may impair myocardial energy metabolism while increasing macrophage dependence on glycolysis and promoting inflammatory metabolic reprogramming (Wan et al., 2025). Previous studies have shown that modulating immunometabolism in inflammatory macrophages can attenuate heart transplant rejection and persistent inflammatory injury (Chen et al., 2024). Consistent with these findings, glycolytic activity was increased in DOX-stimulated pro-inflammatory macrophages in the present study, suggesting that macrophage glycolysis may contribute to the inflammatory component of DOX-induced cardiotoxicity. Nevertheless, the specific metabolic pathways regulating CCR2^+^ macrophage function remain to be fully defined.

PKM2 is a glycolytic enzyme implicated in the metabolic adaptation of pro-inflammatory macrophages. In our study, changes in PKM2 expression and phosphorylation accompanied changes in glycolytic activity, but the causal contribution of PKM2 remains uncertain. Phosphorylation of PKM2 at serine 37 facilitates its nuclear translocation and has been linked to the metabolic reprogramming of pro-inflammatory macrophages in atherosclerosis (Doddapattar et al., 2022). These findings raise the possibility that PKM2-related signaling may modulate macrophage phenotype through glycolytic metabolism. Macrophage-specific PKM2 loss-of-function or rescue experiments will be required to test this possibility.

Shikonin is a naturally occurring naphthoquinone compound derived from Lithospermum erythrorhizon and Arnebia euchroma and has anti-inflammatory, antioxidant, antibacterial, and cytotoxic activities (Guo et al., 2019). Previous studies have linked Shikonin to macrophage metabolic regulation by inhibiting PKM2 dimerization or activity, suppressing aerobic glycolytic reprogramming, and reducing inflammatory macrophage activation in experimental colitis (Huang et al., 2022). Another study reported that Shikonin alleviated DOX-induced cardiac injury through the Mst1/Nrf2 pathway and reduction of oxidative stress and inflammation (Tuo et al., 2024). Compared with these studies, the present work focuses on CCR2^+^ MHC II^high^ macrophages and suggests a potential mechanism involving PKM2 phosphorylation, glycolytic activity, and macrophage–cardiomyocyte crosstalk.

Because Shikonin is a pleiotropic compound, these mechanisms should be considered complementary rather than mutually exclusive. Its cardioprotective effects cannot be attributed exclusively to PKM2 inhibition and may also involve effects on oxidative stress, inflammatory signaling, mitochondrial function, or cardiomyocyte survival. Although our findings are consistent with an association between Shikonin treatment, reduced PKM2 phosphorylation, and macrophage metabolic reprogramming, the relative contributions of PKM2-dependent and PKM2-independent mechanisms remain to be determined.

Several issues must be addressed before clinical translation. The doses and administration route used in mice cannot be directly extrapolated to humans, and the pharmacokinetics, tissue distribution, bioavailability, metabolism, and therapeutic window of Shikonin during DOX chemotherapy remain insufficiently defined. Its poor solubility may further limit systemic exposure and *in vivo* reproducibility, highlighting the need for formulation and pharmacokinetic studies. In addition, any cardioprotective intervention must preserve the antitumor efficacy of DOX. Because Shikonin has cytotoxic and antiproliferative properties, its effects on different tumor types, including DLBCL, as well as on DOX pharmacokinetics and tissue distribution, should be evaluated in tumor-bearing models. Macrophage metabolic modulation may also have context-dependent effects on antitumor immunity.

Finally, the safety of Shikonin requires systematic evaluation. Its pleiotropic activities may affect immune-cell function, hematopoiesis, liver and kidney function, reproductive tissues, and other organs. The safety of chronic Shikonin administration in combination with DOX, including its effects on immune surveillance, infection susceptibility, wound healing, and chemotherapy-related hematological toxicity, remains unknown. Overall, our findings support further preclinical investigation of CCR2^+^ macrophage metabolism and Shikonin-based cardioprotection but do not yet establish Shikonin as a clinically applicable cardioprotective therapy.

## 5 Limitations

Several limitations of this study should be acknowledged. First, the human cohort was relatively small and exploratory. All patients with DLBCL received doxorubicin-containing R-CHOP chemotherapy rather than doxorubicin monotherapy. Therefore, the observed increases in circulating CCR2^+^ MHC II^high^ macrophages, changes in BNP and CRP, and alterations in diastolic parameters cannot be attributed solely to doxorubicin. Cyclophosphamide, vincristine, rituximab, prednisone, disease-related inflammation, cancer stage, disease burden, cumulative doxorubicin exposure, and other clinical factors may independently influence immune-cell composition, inflammatory biomarkers, or cardiac function. Patients with a history of heart failure, active infection, autoimmune disease, clinically significant hepatic or renal dysfunction, or concomitant treatment with other immunomodulatory agents were excluded to reduce major sources of clinical heterogeneity; however, residual confounding cannot be excluded. Because of the limited sample size, reliable multivariable adjustment and subgroup analyses were not feasible.

In addition, the three healthy controls were not age-matched to the patients. The controls were 24, 33, and 35 years old, whereas the patient cohort ranged from 26 to 72 years of age. Age-related differences in circulating immune-cell composition and diastolic cardiac function may therefore have contributed to the observed patient–control differences, and reliable age adjustment was not feasible. Accordingly, the human findings should be interpreted as associative and hypothesis-generating rather than as evidence specifically attributable to doxorubicin.

Second, the animal experiments were conducted in male mice over a relatively limited observation period. Sex-related differences, long-term cardiac remodeling, and delayed cardiotoxicity were therefore not fully assessed. In addition, some cardiac flow-cytometric analyses required pooling hearts from two mice, which reduced the number of independent biological samples and limited the precision of subgroup analyses.

Third, the *in vitro* experiments used RAW264.7 macrophages and H9C2 cardiomyoblasts. These cell lines do not fully recapitulate the biology of primary human cardiac macrophages, human cardiomyocytes, or the complex multicellular cardiac microenvironment. Further studies using primary cells, human induced pluripotent stem cell-derived cardiomyocytes, and more physiologically relevant co-culture or organoid models will be important for validation.

Fourth, although our findings are consistent with a potential role for PKM2-related metabolic reprogramming in the pro-inflammatory phenotype of CCR2^+^ macrophages, macrophage-specific PKM2 knockout, knockdown, overexpression, or rescue experiments were not performed. Thus, the present study cannot establish whether PKM2 is necessary or sufficient for macrophage phenotypic switching or for the cardioprotective effects observed with Shikonin. Moreover, Shikonin has multiple biological activities, including anti-inflammatory, antioxidant, mitochondrial, and cytotoxic effects. The observed reduction in macrophage activation and cardiac injury may therefore involve both PKM2-related and PKM2-independent mechanisms. The present pharmacological approach cannot fully exclude effects involving NF-κB signaling, oxidative stress, mitochondrial function, or other molecular targets. Future studies should combine macrophage-specific genetic manipulation of PKM2 with structurally unrelated PKM2 inhibitors, appropriate rescue experiments, and pathway-specific controls to define the contribution of PKM2 more precisely.

Fifth, the translational feasibility of Shikonin remains uncertain. Its pharmacokinetics, bioavailability, tissue distribution, optimal dose, treatment schedule, and potential interactions with doxorubicin and other components of R-CHOP chemotherapy require further investigation. It will also be important to determine whether Shikonin preserves the antitumor efficacy of doxorubicin while reducing cardiotoxicity. In addition, no formal *a priori* power calculation was performed because this was an exploratory mechanistic study. Several experiments had relatively small sample sizes, and distributional assumptions were not systematically assessed for every dataset. Therefore, the present results should be considered exploratory and hypothesis-generating. Larger confirmatory studies with prospective power calculations, prespecified primary endpoints, systematic assessment of distributional assumptions, and a predefined multiplicity-control strategy are warranted.

Finally, this study was not designed to establish the clinical safety of Shikonin. Although no major adverse phenotype was observed during the short-term mouse experiments, comprehensive assessments of hepatic, renal, hematological, immune, reproductive, and long-term toxicity were not performed. Dedicated pharmacokinetic and toxicological studies will therefore be required before clinical translation.

## 6 Conclusions

Our findings support a contributory role of cardiac CCR2^+^ macrophages in doxorubicin-induced myocardial injury and suggest that PKM2-associated metabolic reprogramming may contribute to the pro-inflammatory phenotype of these cells. Shikonin attenuated macrophage inflammatory features and cardiac injury parameters in the present experimental models. However, because Shikonin has multiple biological activities, including anti-inflammatory, antioxidant, mitochondrial, and cytotoxic effects, its protective effects cannot be attributed exclusively to PKM2 inhibition. Further studies using macrophage-specific PKM2 genetic manipulation, structurally unrelated PKM2 inhibitors, and appropriate pathway-specific controls are required to define the contribution of PKM2 and other mechanisms.

## Data Availability

The original contributions presented in the study are included in the article/**Supplementary Material**. Further inquiries can be directed to the corresponding author.
