# Supplementary material for: Metabolic reprogramming of CCR2+ MHC IIhigh macrophage contributes to myocarditis induced by Doxorubicin
Source: Front Physiol. 2026 Oct 27;17:1883296. doi: 10.3389/fphys.2026.1883296 (PMC13747856; doi:10.3389/fphys.2026.1883296)
Supplement: Supplementary file 1 [file Supplementaryfile1.docx]

**Supplementary Table**

**Supplementary Table 1. Information of patients receiving chemotherapy and normal control**

|  | Sex | Age | Chemotherapy strategy | BNP (pg/mL) | CRP  (mg/L) | ECG changes | Systolic Function (EF, %) | Diastolic  Function (E/A) | Pericardial effusion |
| --- | --- | --- | --- | --- | --- | --- | --- | --- | --- |
| Patient 1 | Female | 63 | Traditional Doxorubicin | 165.9 | 79.04 | (－) | 60 | ＜1 | (+) |
| Patient 2 | Female | 70 | Traditional Doxorubicin | 233.7 | 48.32 | (－) | 59 | ＜1 | (－) |
| Patient 3 | Male | 55 | Traditional Doxorubicin | ＜10 | 23.51 | (+)  Sinus tachycardia | 62 | ＜1 | (－) |
| Patient 4 | Male | 26 | Traditional Doxorubicin | ＜10 | 14.66 | (+)  Sinus tachycardia | 65 | ＜1 | (－) |
| Patient 5 | Female | 46 | Traditional Doxorubicin | 20.4 | 18.42 | (+)  Complete right bundle branch block and T wave changes | 62 | ＜1 | (－) |
| Patient 6 | Female | 62 | Liposomal Doxorubicin | 27.3 | 8.86 | (+)  T wave changes | 65 | ＜1 | (－) |
| Patient 7 | Male | 67 | Liposomal Doxorubicin | 22 | 11.2 | (－) | 65 | ＞1 | (－) |
| Patient 8 | Female | 59 | Liposomal Doxorubicin | 33.5 | 18.49 | (－) | 65 | ＜1 | (－) |
| Patient 9 | Female | 72 | Liposomal Doxorubicin | 117.5 | 0.95 | (+)  Frequent premature ventricular contractions | 63 | ＜1 | (－) |
| Patient 10 | Male | 72 | Liposomal Doxorubicin | 344.3 | 116.63 | (+)  Frequent atrial premature beats | 62 | ＜1 | (－) |
| Control 1 | Female | 24 | / | / | / | / | / |  |  |
| Control 2 | Male | 33 | / | / | / | / | / |  |  |
| Control 3 | Female | 35 | / | / | / | / | / |  |  |

**Supplementary Table 2. Exclusion criteria**

| Characteristic | Patient cohort |
| --- | --- |
| Number of patients | n = 10 |
| Age, years | 26 - 72 |
| Male sex, n (%) | 40 |
| Diagnosis | DLBCL |
| Chemotherapy regimen | R-CHOP |
| Doxorubicin-containing regimen | Yes |
| History of heart failure | Excluded |
| Active infection | Excluded |
| Autoimmune disease | Excluded |
| Significant hepatic dysfunction | Excluded |
| Significant renal dysfunction | Excluded |
| Concomitant immunomodulatory treatment | Excluded |

*Note.* Patients with a history of heart failure, active infection, autoimmune disease, clinically significant hepatic or renal dysfunction, or concomitant immunomodulatory treatment were excluded from the clinical cohort; All patients had a confirmed diagnosis of DLBCL and received doxorubicin-containing R-CHOP chemotherapy, consisting of rituximab, cyclophosphamide, doxorubicin, vincristine, and prednisone. Doxorubicin was not administered as monotherapy in this cohort.

**Supplementary Table 3. FACS Information of splenic and cardiac immune cells**

| **FACS Information of splenic immune cells** | | |
| --- | --- | --- |
| Fluorochromes | FACS Antigens | ID |
| BV-510 | Zombie Aqua™ Fixable Viability Kit | Biolegend: 423101 |
| FITC | anti-mouse CD45 | Biolegend: 103108 |
| PE/Cyanine7 | anti-mouse/human CD11b | Biolegend: 101215 |
| PE | anti-mouse F4/80 | Biolegend: 123109 |
| **FACS Information of cardiac immune cells** | | |
| Fluorochromes | FACS Antigens | ID |
| BV-510 | Zombie Aqua™ Fixable Viability Kit | Biolegend: 423101 |
| FITC | Anti-mouse CD45 | Biolegend: 103108 |
| PE/Cyanine7 | Anti-mouse/human CD11b | Biolegend: 101215 |
| PE | Anti-mouse F4/80 | Biolegend: 123109 |
| Alexa Fluor® 647 | Anti-mouse CD192 (CCR2) | Biolegend:150603 |
| PerCP/Cyanine5.5 | Anti-mouse I-A/I-E (MHC II) | Biolegend: 107625 |

**Supplementary Table 4. Sequences of the primers for real time RT-PCR:**

| Gens | primers | sequences |
| --- | --- | --- |
| IL-6 | Forward  Reverse | 5´- ACAACCACGGCCTTCCCTACT -3´  5´- CTCATTTCCACGATTTCCCAGA -3´ |
| IL-1β | Forward  Reverse | 5´- AAATACCTGTGGCCTTGGGC -3´  5´- CTTGGGATCCACACTCTCCAG -3´ |
| PKM2 | Forward  Reverse | 5´- TGTCTGGAGAAACAGCCAAG -3´  5´- TCCTCGAATAGCTGCAAGTG -3´ |
| HK2 | Forward  Reverse | 5´- TGATCGCCTGCTTATTCACGG -3´  5´- AACCGCCTAGAAATCTCCAGA -3´ |
| GLUT1 | Forward  Reverse | 5´- CATCGTGGCCATCTTTGGCTTTGT -3´  5´- GGAAGCACATGCCCACAATGAAGT -3´ |
| Ldha | Forward  Reverse | 5´- TGTCTCCAGCAAAGACTACTGT -3´  5´- GACTGTACTTGACAATGTTGGGA -3´ |
| ALDOA | Forward  Reverse | 5´- AGTCCACCGGAAGCATTGC -3´  5´- CAGCCCCTGGGTAGTTGTC -3´ |
| PFK2 | Forward  Reverse | 5´- GGTCCGAGTTGGTATCTTCAC -3´  5´- ACTTCCAATCACTGTGCCAC -3´ |
| β-Actin | Forward  Reverse | 5´- GGCTGTATTCCCCTCCATCG -3´  5´- CCAGTTGGTAACAATGCCATGT -3´ |

**Supplementary Table 5. Antibodies used in this study**

| Target protein | Catalog number | Manufacturer | Reference |
| --- | --- | --- | --- |
| STAT3 | 10253-2-AP | Proteintech | 39515328 |
| P-STAT3 (Tyr705) | 9145 | Cell Signaling Technology | 42559137 |
| PKM2 | 15822-1-AP | Proteintech | 18337823 |
| P-PKM2 (Ser37) | AF7231 | Affinite | 41145464 |
| β-actin | 66009-1-Ig | Proteintech | 33278357 |

**Supplementary Table 6. Echocardiographic analysis of each group on day 0, day 7, day 14, day 21, and Shikonin (SHI)treatment**

|  | Heart rate (bpm) | Ejection fraction (%) | Fractional shortening (%) | LVAW, s  (mm) | LVAW, d  (mm) |
| --- | --- | --- | --- | --- | --- |
| Day 0 | 365.5±10.8 | 66.8±3.2 | 35.8±2.4 | 1.3±0.1 | 0.9±0.1 |
| Day 7 | 457.6±85.7^*^ | 56.5±3.0^***^ | 29.0±1.8^***^ | 0.9±0.1^*^ | 0.7±0.1 |
| Day 14 | 341±37.6 | 56.9±4.0^***^ | 29.0±2.4^***^ | 1.0±0.3 | 0.9±0.4 |
| Day 21 | 385.8±10.3 | 55.8±1.7^***^ | 28.9±1.4^***^ | 0.8±0.1^**^ | 0.6±0.2 |
| SHI 2mg/Kg | 381±33.3 | 64.1±5.2^##^ | 36.1±1.5^###^ | 1.1±0.1 | 0.7±0.1 |
| SHI 5mg/Kg | 358±31.0 | 65.6±4.6^##^ | 35.2±3.3^###^ | 1.1±0.1^#^ | 0.8±0.07 |

Error bars show mean ± SD. P values were determined by one-way ANOVA followed by Tukey’s post hoc test. ESV: end-systolic volume. LVAW, s: Left ventricular anterior wall thickness in diastole. LVAW, d: Left ventricular posterior wall thickness in systole. *^*^P < 0.05* compared to Control, *^**^P < 0.01* compared to Control, *^***^P < 0.001* compared to Control. *^#^P < 0.05* compared to Day 21, *^##^P < 0.01* compared to Day 21, *^###^P < 0.001* compared to Day 21.

**Supplementary Figure**

**Supplementary Figure 1. Flow cytometry gating strategy of patients’ blood samples**

**Supplementary Figure 2. Flow cytometry gating strategy of cardiac immune cell subsets**

**Supplementary Figure 3. Flow cytometry gating strategy of splenic immune cell subsets**

**Supplementary Figure 4. Effects of shikonin on liver function in mice**

Data were shown as mean ± SD. One-way analysis of variance for comparison of multiple groups, following by Tukey’s multiple comparison tests. *ns*, not statistically significant (*P > 0.05*).

**Supplementary Figure 5. Western blotting of PNMCs was performed to identify total phosphorylated (Tyr 705) levels of STAT3 in different group**
